# The Involvement of RIPK1 in Alopecia Areata

**DOI:** 10.3390/ijms26041565

**Published:** 2025-02-13

**Authors:** Hyunju Kim, Mei Zheng, Seungchan An, In Guk Park, Leegu Song, Minsoo Noh, Jong-Hyuk Sung

**Affiliations:** 1 Epi Biotech Co., Ltd., Incheon 21983, Republic of Korea; hjkim@epibiotech.com (H.K.); mzheng@epibiotech.com (M.Z.); lgsong@epibiotech.com (L.S.); 2 Natural Products Research Institute, College of Pharmacy, Seoul National University, Seoul 08826, Republic of Korea; ann081993@snu.ac.kr (S.A.); ingukpark@snu.ac.kr (I.G.P.); 3 College of Humanities, Interdisciplinary Program in Cognitive Science, Seoul National University, Seoul 08826, Republic of Korea

**Keywords:** RIPK1, alopecia areata, dendritic cell, CD8^+^ T cell, Necrostatin-1s, GSK2982772

## Abstract

We have previously demonstrated that receptor-interacting serine threonine kinase 1 (RIPK1) is expressed in hair follicles and regulates the hair cycle. In a mouse model, RIPK1 inhibitors also accelerated the telogen-to-anagen transition and elongated the anagen period. Here, we first investigated the involvement of RIPK1 in alopecia areata (AA). The mRNA and protein expression of RIPK1 was increased in the skin of an AA mouse model. Single-cell RNA sequencing and immunohistochemistry showed that RIPK1 was highly increased in dendritic cells (DCs) and CD8^+^ T cells. RIPK1 inhibitors (i.e., Necrostatin-1s and GSK2982772) delayed the onset of AA in the mouse model and reduced the numbers of DCs and CD8^+^ T cells in AA skin. The RIPK1 inhibitors also increased the hair length in a mouse hair organ culture mimicking AA. Collectively, these results suggest that RIPK1 is involved in AA onset via modulating immune cells, and RIPK1 inhibitors could prevent AA onset.

## 1. Introduction

Receptor-interacting serine/threonine kinase 1 (RIPK1) is a critical regulator of the cell death pathway, particularly necroptosis, and plays a central role in various inflammatory processes [1]. RIPK1 is activated downstream of tumor necrosis factor receptor 1 (TNFR1) and Toll-like receptors (TLRs), and is also involved in interferon signaling [2]. As a multifunctional protein, RIPK1 is crucial for maintaining cellular homeostasis and regulating programmed cell death. Its dysregulation has been implicated in numerous inflammatory diseases, including neurodegenerative and autoimmune disorders [1]. RIPK1 is also involved in skin inflammation, and the activation of RIPK1 is reportedly correlated with many skin diseases, including melanoma, psoriasis, systemic lupus erythematosus, and hair loss [3,4].

In hair biology, the role of the cell death pathway, including apoptosis, autophagy, and necroptosis, is critical for hair follicle (HF) cycling and regeneration. Apoptosis plays a key role in HF regression during the catagen phage, while autophagy is linked to hair growth and HF activation during the anagen phase [5,6,7,8]. However, in comparison to apoptosis and autophagy, the involvement of necroptosis or RIPK1 expression during the hair cycle progression is less clear. Our recent study indicated that RIPK1 is highly expressed in the outer root sheath (ORS) during the HF regression period, suggesting that necroptosis may be involved in hair cycle progression [4].

Interestingly, RIPK1 inhibitors such as Necrostatin-1s (Nec-1s) induced ORS cell proliferation and migration and increased the HF length in mouse and pig organ cultures. In addition, RIPK1 inhibitors enhanced the telogen-to-anagen transition in an animal model [4]. Recently, Jang et al. sought to determine whether necroptosis is associated with the pathogenesis of alopecia areata (AA); however, the mRNA and protein expression of RIPK1 and RIPK3 was not upregulated in the skin lesions of patients with AA [9]. In contrast, we prepared an AA mouse model via lymph node cell injection and performed single-cell RNA sequencing (scRNA-seq), finding that the mRNA level of *Ripk1* was increased. Therefore, in the present study, we sought to determine whether RIPK1 is involved in AA pathology and whether RIPK1 inhibitors could attenuate AA progression in an AA animal model.

## 2. Results

### 2.1. Increased Expression of Ripk1 in AA Mouse Skin

**Figure 1:**
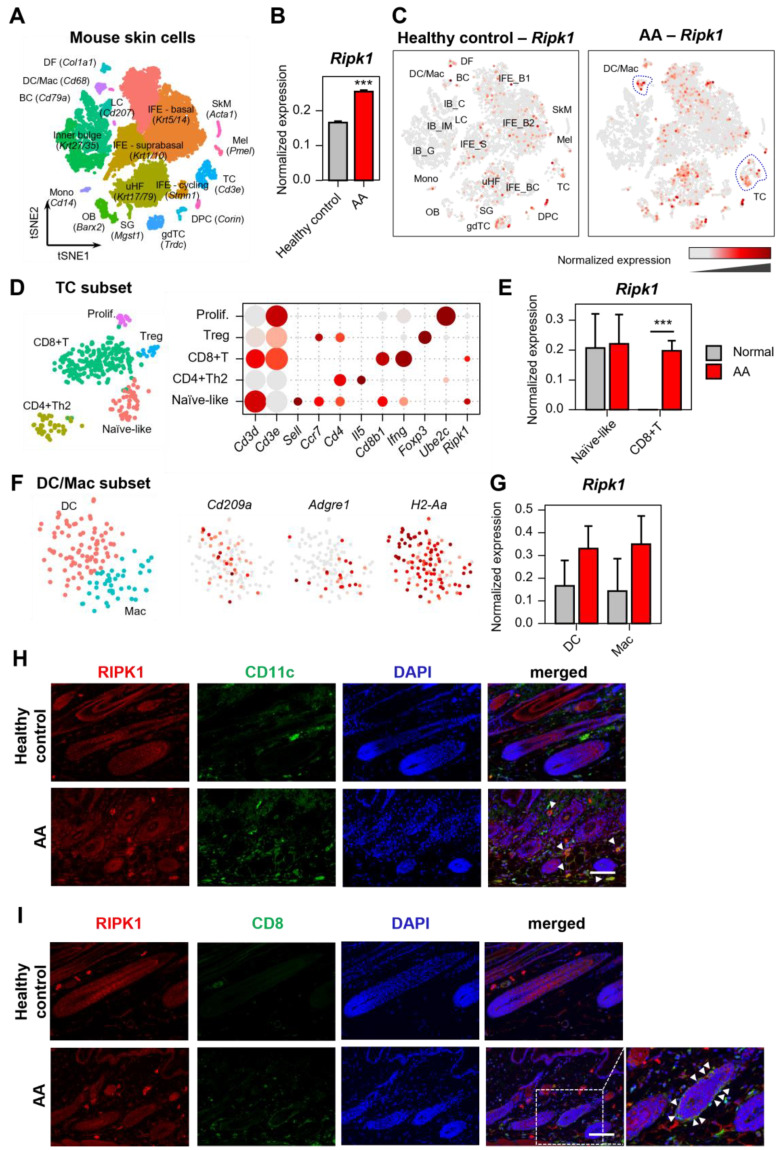
Expression profile of *Ripk1* in the AA mouse model. (**A**) tSNE plot showing the clustering of mouse skin cell types based on scRNA-seq data. Cell type annotations were assigned using marker genes indicated in parentheses. (**B**) Comparison of normalized *Ripk1* expression in healthy control and AA mouse samples. *** *p* < 0.001. (**C**) tSNE visualization of *Ripk1* expression in healthy control (left) and AA (right) mouse skin cells. Blue dotted circles indicate the DC/Mac and T cell clusters. (**D**) Identification of T cell subpopulation. The dot plot presents marker gene expression in naïve-like, CD4+ T helper 2 (Th2), CD8+ T, regulatory T cells (Treg), and proliferating T cells (Prolif). Dot size indicates the proportion of cells in each group. (**E**) Normalized expression of *Ripk1* in T cell subsets. *** *p* < 0.001. (**F**) Identification of DC/Mac subpopulation. (**G**) Normalized expression of *Ripk1* in DC and macrophage. (**H**) Representative images from the skin sections of healthy control and AA mice after immunostaining with RIPK1, CD11c, and DAPI for nuclei. White arrows point to DCs expressing RIPK1. Scale bar: 100 μm. (**I**) Representative images from the skin sections of healthy control and AA mice after immunostaining with RIPK1, CD8, and DAPI for nuclei. Scale bar: 100 μm. The enlarged region within the white dotted box is shown on the right. White arrows point to CD8^+^ T cells expressing RIPK1.

The C3H/HeN mouse model of AA was generated by transplanting skin-draining lymph node (SDLN) cells from an AA donor into a naïve C3H/HeN recipient. This model demonstrates strong concordance with human AA and has been widely used in translational studies [10,11,12,13]. To comprehensively profile the mechanism underlying AA, we performed droplet-based scRNA-seq on single cells isolated from mice with AA and control C3H/HeN. We obtained the transcriptomic profiles of 14,469 cells that passed the primary quality control filter. Using t-distributed stochastic neighbor embedding (t-SNE) and a subsequent clustering approach, we identified 20 cell clusters from the scRNA-seq data (Figure 1A).

Interestingly, the expression level of the *Ripk1* gene in AA was higher than in the control mice without hair loss, which was demonstrated by pseudobulk analysis (Figure 1B). Further analysis showed a notable increase in *Ripk1* expression within specific immune cell subtypes, particularly T cell (TC) and dendritic cell/macrophage (DC/Mac) subpopulations within the immune cell groups (Figure 1C). To determine which TC subtypes expressed *Ripk1*, we analyzed the TC subpopulations, identifying five distinct subsets: naïve-like, Th2, CD8+, Treg, and proliferating TC (Figure 1D). Among these, *Ripk1* expression was significantly elevated in CD8+ T cells of AA mice compared to normal controls (Figure 1E). Within the DC/Mac population, *Cd209a* and *Adgre1* expression markers were used to distinguish DCs from macrophages (Figure 1F). Although the increase in *Ripk1* expression within these populations was not statistically significant, it showed a substantial difference (Figure 1G).

To validate this finding, we further observed the increased protein expression of RIPK1 in AA skin. As expected, RIPK1 is co-expressed and increased in DC marker (CD11c) and CD8^+^ T cell marker (CD8), suggesting that RIPK1 is up-regulated in the immune cells of AA skin (Figure 1H,I).

However, the *Ripk3* and *Mlkl* mRNA expression was very low in AA mouse skin (Figure S1), suggesting that necroptosis might not be involved in AA progression. Collectively, these results suggest that RIPK1 is up-regulated in immune cells, plays a role in immune cell infiltration, and contributes to AA progression.

### 2.2. Prevention of AA Onset by Nec-1s

**Figure 2:**
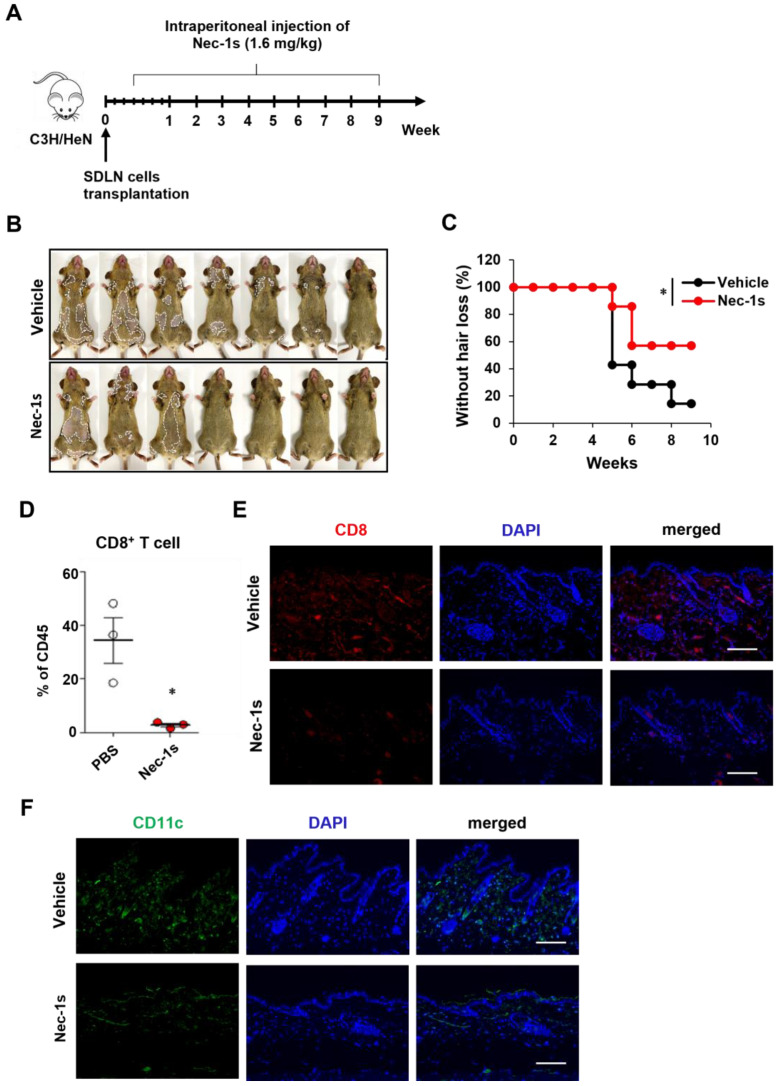
RIPK1 inhibition with Nec-1s prevents the onset of AA in C3H/HeN mice. (**A**) Design of animal experiment. C3H/HeN female mice were treated with Nec-1s (1.6 mg/kg) through intraperitoneal injection daily for 9 weeks, starting 3 days after AA induction. (**B**) Representative images of individual Nec-1s- or vehicle-treated C3H/HeN mice after 9 weeks of treatment. The areas of hair loss are marked in the images with dotted white lines. (**C**) Time course of hair loss shown after Nec-1s treatment. * *p* < 0.05. (**D**) Percentage of CD8^+^ T cells within skin cell suspensions analyzed via flow cytometry. * *p* < 0.05. (**E**) Representative immunofluorescence image of skin section stained with anti-CD8 antibody and DAPI for nuclei. Scale bar: 100 μm. (**F**) Representative immunofluorescence image of skin section stained with anti-CD11c antibody and DAPI for nuclei. Scale bar: 100 μm. This experiment was performed for 7 mice per group.

We investigated the role of RIPK1 in AA pathogenesis through an RIPK1 signaling blockade. Nec-1s has been used to inhibit RIPK1 activation when administrated in vivo [14,15]. Here, C3H/HeN mice were treated with Nec-1s beginning three days after the transplantation of allogenic SDLN cells to induce AA (Figure 2A). The Nec-1s-treated mice showed a reduction in the number of AA-affected mice compared to the control group (Figure 2B,C). The role of RIPK1 in AA development was also confirmed by comparing the quantities of skin-infiltrating CD8^+^ T cells. The flow cytometry analysis of skin cell suspensions revealed that the number of CD8^+^ T cells among skin CD45^+^ immune cells was significantly reduced in the Nec-1s-treated mice (Figure 2D). Consistent with the FACS results, Nec-1s substantially reduced the amount of the CD8 stained cells in the skin (Figure 2E). We next analyzed the changes in the DC amount, based on CD11c staining in the skin. The Nec-1s treatment significantly reduced the number of DCs compared to the control (Figure 2F). Next, we analyzed interlukin-15 (IL-15) and interferon-γ (IFN-γ), as the expression levels of IL-15 and IFN-γ play a significant role in AA pathogenesis. Elevated levels of these cytokines have been observed in affected scalp regions, suggesting their involvement in the progression and severity of AA [16]. As expected, Nec-1s treatment significantly reduced the expression of IL-15 and IFN-γ in the AA mice (Figure S2A). These results suggest that RIPK1 inhibition could alleviate AA by preventing inflammation and immune cell infiltration.

### 2.3. Prevention of AA Onset by GSK2982772

**Figure 3:**
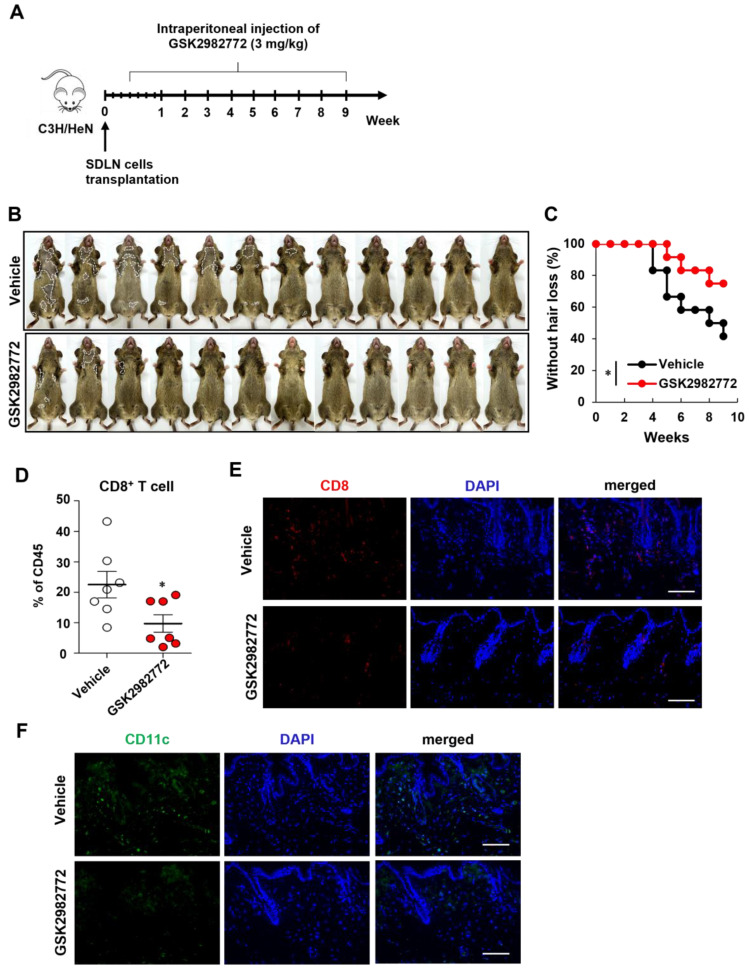
RIPK1 inhibition by GSK2982772 prevents the onset of AA in C3H/HeN mice. (**A**) Design of animal experiment. C3H/NeH female mice were treated with GSK2982772 (3 mg/kg) through intraperitoneal injection daily for 9 weeks, starting 3 days after AA induction. (**B**) Representative images of individual GSK2982772- or vehicle-treated C3H/HeN mice after 9 weeks of treatment. The areas of hair loss are marked in the images with dotted white lines. (**C**) Time course of hair loss shown after GSK2982772 treatment. * *p* < 0.05. (**D**) Percentage of CD8^+^ T cells within skin cell suspensions analyzed via flow cytometry. * *p* < 0.05. (**E**) Representative immunofluorescence image of skin section stained with anti-CD8 antibody and DAPI for nuclei. Scale bar: 100 μm. (**F**) Representative immunofluorescence image of skin section stained with anti-CD11c antibody and DAPI for nuclei. Scale bar: 100 μm. This experiment was performed for 12 mice per group.

We also investigated the preventive effects of another RIPK1 inhibitor (GSK2982772). As expected, the GSK2982772-treated mice showed reduction in the number of AA-affected mice compared to the control mice (Figure 3B,C). The flow cytometry analysis of skin cell suspensions also revealed that the number of CD8^+^ T cells among the skin CD45^+^ immune cells was significantly reduced in the GSK2982772-treated mice (Figure 3D). GSK2982772 substantially reduced the amount of the CD8 stained cells in the skin (Figure 3E). The GSK2982772 treatment also significantly reduced the number of DCs compared to the control (Figure 3F). In addition, GSK2982772 reduced the expression of IL-15 and IFN-γ in the AA mice (Figure S2B).

### 2.4. Decreased AA-like Symptoms in Mouse Vibrissa Follicles Due to RIPK1 Inhibitors

**Figure 4:**
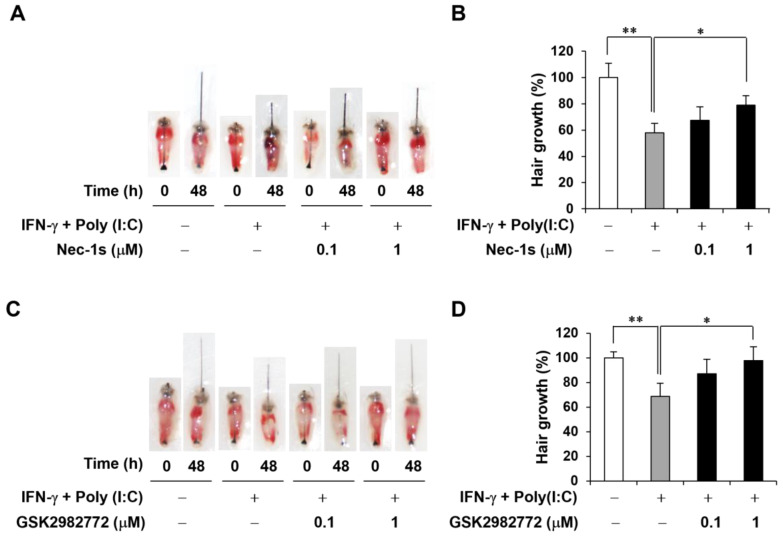
RIPK1 inhibitors increased hair shaft growth in IFN-γ- and poly(I:C)-treated mouse vibrissa follicles. (**A**) Images of follicles were taken after 0 or 48 h Nec-1s (0.1 or 1 μM) treatment in IFN-γ- and poly(I:C)-induced AA model, respectively. (**B**) Nec-1s increased hair length in ex vivo AA model. (**C**) Images of follicles treated with GSK2982772 (0.1 or 1 μM) in AA model. (**D**) GSK2982772 increased hair length in ex vivo AA model. * *p* < 0.05, ** *p* < 0.01.

It has been reported that IFN-γ and polyinosinic–polycytidylic acid [poly(I:C)] induce AA-mimicking conditions, and we established an ex vivo mouse vibrissa organ culture model [17]. We then investigated whether RIPK1 inhibitor treatment would affect the length of the mouse vibrissa follicle. The mouse vibrissa follicles had been isolated and cultured with IFN-γ and poly(I:C) with RIPK1 inhibitors for 48 h. Images of the follicles were taken at 0 and 48 h (Figure 4A,C). As expected, IFN-γ and poly(I:C) induction retarded hair shaft growth, but this was attenuated by both analyzed RIPK1 inhibitors (Figure 4B,D).

## 3. Discussion

In the present study, we investigated the involvement of RIPK1 in AA progression. The mRNA and protein expression of RIPK1 was increased in the skin of the AA mouse model. scRNA-seq and immunohistochemistry showed that *Ripk1* was highly increased in DCs and CD8^+^ T cells. RIPK1 inhibitors reduced the onset of AA in the mouse model and reduced the numbers of CD8^+^ T cells and DCs in AA skin. RIPK1 inhibitors also increased the hair length in the mouse hair organ culture. Collectively, these results suggest that RIPK1 is involved in AA onset, and RIPK1 inhibition could prevent AA.

The expression and involvement of RIPK1 in regulating the hair cycle has been demonstrated, and it was found that RIPK1 was upregulated in the hair regression period to induce hair loss [4,18]. However, the involvement of RIPK1 in AA has not been sufficiently reported. Jang et al. sought to determine whether necroptosis was associated with the pathogenesis of AA, but the RIPK1 and RIPK3 mRNA and protein expression was not upregulated in the skin lesions of AA patients [9]. In their study, they collected human scalp skin samples and analyzed whole skin cells, and they did not find differences in necroptosis-related genes. In a mouse AA model, we performed scRNA-seq and found that *Ripk1* expression increased in DCs and T cells, in addition to dermal fibroblasts and keratinocytes. In addition, RIPK1 inhibitors reduced the quantities of immune cells such as DCs and CD8^+^ T cells in AA skin to prevent AA onset. However, the *Ripk3* and *Mlkl* expression was very low in mouse AA skin, suggesting that necroptosis might not be involved in AA progression (Figure S1). On the contrary, RIPK1 might play a key role in AA progression through the infiltration of immune cells such as DCs and CD8^+^ T cells.

AA is due to the loss of immune privilege, leading to an autoimmune attack, and many studies have reported the key immune cells responsible for HF damage [19]. Recently, the functional interrogation of lymphocyte subsets in AA was analyzed in mice and humans using scRNA-seq, and it was found that CD8^+^ T cells were the predominant AA-driving cell type [20]. In this study, AA onset in mice was induced via lymph node cell injection, resulting in significant increases in the number of CD8^+^ T cells, leading to hair loss. Conversely, the injection of RIPK1 inhibitors significantly reduced the number of CD8^+^ T cells in the skin, delaying AA onset. However, RIPK1 inhibitors did not inactivate T cells in response to PHA simulation (Figure S3), which suggests that they do not directly inhibit T cell proliferation.

Chronic inflammatory and autoinflammatory diseases are caused by immune dysregulation, and RIPK1 has emerged as a key regulator of immunity via its integral role in cell death signaling following exposure to inflammatory and infectious stimuli [21]. Therefore, RIPK1 is involved in autoimmune diseases, such as rheumatoid arthritis, psoriasis, and inflammatory bowel disease [22,23,24]. In addition to these autoimmune diseases, for the first time, we demonstrate the involvement of RIPK1 in AA progression and the possibility of applying RIPK1 inhibitors for AA prevention.

## 4. Materials and Methods

### 4.1. Preparation of Single-Cell Suspension

Skin samples were collected from C3H/HeN mice and AA model mice. To ensure a sufficient yield of cells, skin samples from the backs of 7–8 mice per group were pooled, finely minced, and digested with a 0.7 mg/mL collagenase D solution (Sigma, St. Louis, MO, USA). The resulting cell mixture was then filtered through 70 μm and 40 μm meshes, and cellular debris was eliminated via density gradient centrifugation using Percoll media (Sigma).

### 4.2. Single-Cell RNA Sequencing

The single-cell suspensions were processed following the Chromium Next GEM Sin-gle Cell 3′ RNA Library v3.1 protocol (10× Genomics) in accordance with the manufacturer’s guidelines. In brief, individual cells were encapsulated into nanoliter-scale Gel Beads-in-Emulsion (GEMs) that contained barcoded oligonucleotides. The poly(dT) primers within these GEMs selectively captured polyadenylated mRNA from each cell, allowing for the synthesis of barcoded, full-length cDNA. This cDNA was then amplified and used to construct 3′ gene expression libraries, which were sequenced on an Illumina platform at Macrogen. The subsequent analyses, including read alignment to the mouse reference genome (mm10), filtering, barcode counting, and unique molecular identifier (UMI) counting, were performed using the Cell Ranger v7.2.0 (10× Genomics) count pipeline. The sequenced reads have been deposited into the Gene Expression Omnibus (GEO) under accession number GSE269455 [https://www.ncbi.nlm.nih.gov/geo/query/acc.cgi?acc=GSE269455 (accessed on 1 July 2024)].

### 4.3. Single-Cell Transcriptome Analysis

Initial processing was performed using the Seurat v5.0.3 R package, which includes cell filtering, clustering, and annotation, as previously detailed [25,26]. Cells exhibiting over 10% mitochondrial gene expression or expressing fewer than 200 genes were excluded from the analysis, resulting in a total of 14,469 skin cells across two groups (C3H and AA). Potential doublets were identified and removed using DoubletFinder v2.0.4 with default settings [27]. After quality control, the count matrix was normalized, and the 2000 most variable genes were selected for further scaling. Dimensionality reduction was then performed using t-SNE. Cell clusters were manually annotated based on the marker gene expression.

For example, basal interfollicular epidermis (IFE_B) cells were annotated using *Krt5* and *Krt14* as markers, while suprabasal IFE (IFE_S) cells were identified by the expression of *Krt1* and *Krt10*. Cycling basal IFE (IFE_BC) cells were marked by *Krt5*, *Krt14*, *Stmn1*, and *Mki67*. Upper hair follicle (uHF) cells were characterized by *Krt17* and *Krt79*, whereas sebaceous gland (SG) cells were annotated using *Scd1* and *Mgst1*. Outer bulge (OB) cells were identified by the expression of *Barx*. Hair follicle keratinocytes were categorized based on *Krt27* and *Krt35* expression and further subdivided into germinative layer (IB_G), inner root sheath and medulla (IB_IM), and cortex/cuticle (IB_C) cells, depending on the expression of proliferative markers like *Stmn1*.

Fibroblast-like cells were differentiated into dermal fibroblasts (DF) based on *Col1a1* and *Lum* expression, and dermal papilla cells (DPCs) were identified by the expression of *Corin* and *Notum*. Immune cells were classified according to their specific marker genes: T cells (TC, *Cd3e*), gamma-delta T cells (gdTC, *Trdc*), monocytes (Mono, *Cd14*, and *Ccl6*), dendritic cells and macrophages (DC/Mac, *Cd68*, and *Cd74*), Langerhans cells (LC, *Cd207*), and B cells (BC, *Cd79a*). Additionally, skeletal muscle cells (SkM, *Acta1*, and *Des*) and mel-anocytes (*Mel*, *Pmel*, and *Dct*) were also identified based on their characteristic gene ex-pression profiles.

### 4.4. Animals

Male mice aged 4 weeks (vibrissae organ culture) and female mice aged 9 weeks (AA animal model) from the C3H/HeN strain were procured from Orient Bio Co., Ltd. (Sungnam, Republic of Korea). All animal studies were conducted with the approval of the Institutional Animal Care and Use Committee of Yonsei University (IACUC-A-202302-1636-01; approved on: 15 March 2023), adhering to ethical guidelines to minimize suffering and ensure the wellbeing of the animals.

### 4.5. Alopecia Areata Animal Model

The AA mouse model was induced by transferring 1 × 10^7^ in vitro-expanded skin-draining lymph node (SDLN) cells from AA-affected mice, as previously described [28,29]. In brief, SDLN cells were extracted from mice that had spontaneously developed AA and cultured in advanced RPMI 1640 (Gibco, Miami, FL, USA) supplemented with 10% FBS (Gibco), 2 mM GlutaMAX (Gibco), and 100 U/mL penicillin–streptomycin (Hyclone, South Logan, UT, USA). The culture medium was further supplemented with IL-2 (Roche, Basel, Switzerland), IL-7 (R&D Systems, Minneapolis, MN, USA), and IL-15 (R&D Systems). Cells were stimulated using Dynabeads Mouse T-Activator CD3/CD28 (Thermo Fisher Scientific, Waltham, MA, USA) and intradermally transferred to at least 10-week-old C3H/HeN female mice with a normal hair coat during the second telogen phase. AA development was observed starting from 4~5 weeks post-injection.

### 4.6. Flow Cytometry Analysis

Mouse skin tissue was harvested into collagenase type 4 (3 mg/mL, Worthington, Lakewood, NJ, USA) with DNase I (5 μg/mL, Roche) and chopped into small fragments. Samples were incubated at 37 °C for 90 min, then filtered through 70 μm mesh followed by 40 μm mesh. For live/dead staining, the Zombie NIR fixable viability kit (BioLegend, San Diego, CA, USA) was used for 10 min. To block nonspecific binding, cells were incubated with a CD16/CD32 antibody (BD Biosciences, San Jose, CA, USA) for 10 min. For surface staining, the cells were labeled with the following antibodies: FITC anti-CD8 and PerCP-Cy5.5 anti-CD45 (BioLegend). Samples were acquired using CytoFLEX (Beckman Coulter, Miami, FL, USA), and the data were analyzed with CytExpert software (version 2.4.0.28).

### 4.7. Immunostaining

For immunofluorescence studies of mouse skin, 5 μM formalin fixed and paraffin skin section were used. After heating antigen retrieval, skin sections were stained with the following primary antibodies: anti-RIPK1 (Novus Biologicals, Centennial, CO, USA), anti-CD11c (Santa Cruze Biotechnology, Dallas, TX, USA), anti-CD8 (Santa Cruze Biotechnology), anti-IL-15 (R&D Systems), and anti-IFN-γ (ABclonal, Wuhan, China). Alexa Fluor 488- or Alexa Fluor 594-conjugated goat anti-Rabbit, goat anti-Mouse, or donkey anti-Goat antibody was used as a secondary antibody (Invitrogen, Carlsbad, CA, USA). Immunofluorescence images were captured using a Nikon Eclipse Ts2 microscope (Nikon, Tokyo, Japan).

### 4.8. Hair Organ Culture

The hair growth activity of mouse vibrissae was observed in organ culture, which follows the method previously described by Jindo and Tsuboi [30]. Normal anagen vibrissae HFs were obtained from the upper lip region using a scalpel and forceps. Then, the isolated HFs were placed in a defined medium (Williams E medium supplemented with 2 mM L-glutamine, 10 µg/mL insulin, 10 ng/mL hydrocortisone, 100 U/mL penicillin, and 100 μg/mL streptomycin, without serum). Individual vibrissa HFs were photographed 48 h after the start of the incubation. Changes in hair length were calculated from these photographs and expressed as the mean ± standard error (SE) of 10–12 vibrissae HFs.

### 4.9. Statistical Analysis

All data are demonstrated as the mean ± standard deviations from at least three independent experiments. For the analysis between two groups, Student’s *t*-test was used. For analysis of hair loss curves, Log-rank tests were used. When more than two groups were compared, a one-way analysis of variance (ANOVA) test followed by Tukey’s post hoc test was applied. The significance values were set as follows: * *p* < 0.05, ** *p* < 0.01, and *** *p* < 0.001. All statistical analyses were performed using GraphPad Prism 5.01 (GraphPad Software Inc., San Diego, CA, USA).

## Data Availability

The data generated during the current study are openly available in the GEO repository with GEO accession number GSE269455 (https://www.ncbi.nlm.nih.gov/geo/query/acc.cgi?acc=GSE269455, accessed on 26 August 2024).

## Supplementary Materials

The supporting information can be downloaded at https://www.mdpi.com/article/10.3390/ijms26041565/s1.
