# Supplementary material for: The Involvement of RIPK1 in Alopecia Areata
Source: Int J Mol Sci. 2025 Feb 13;26(4):1565. doi: 10.3390/ijms26041565 (PMC11855397; doi:10.3390/ijms26041565)
Supplement: Supplementary file 1 [file ijms-26-01565-s001.zip › ijms-3442900-supplementary.pdf]

Article

The Involvement of RIPK1 in Alopecia Areata

Hyunju Kim [1], Mei Zheng [1], Seungchan An [2], In Guk Park [2], Leegu Song [1,3], Minsoo Noh [2,*] and Jong-Hyuk Sung [1,*]

[1] Epi Biotech Co., Ltd., Incheon 21983, Republic of Korea
[2] Natural Products Research Institute, College of Pharmacy, Seoul National University, Seoul 08826, Republic of Korea
[3] College of Humanities, Interdisciplinary Program in Cognitive Science, Seoul National University, Seoul 08826, Republic of Korea
* Correspondence: minsoonoh@snu.ac.kr (M.N.); brian99@epibiotech.com (J.-H.S.); Tel.: +82-02-880-2481 (M.N.); +82-70-4209-0556 (J.-H.S.)

1. Materials and Methods

1.1. Stimulation of cells with PHA and cell proliferation ELISA, BrdU assay

PBMCs were cultured at a density of 1×10^5 cells/well in a 96-well plate in the presence of different concentrations of Nec-1s (0.01, 0.1, 1, 10 μM). For PHA stimulation, PHA (1 μg/ml) was added to the culture medium 30 min after Nec-1s addition, and the cells were incubated for 48 h. Relative proliferation was determined via the cell proliferation ELISA, BrdU assay (Roche, Rotkreuz, Switzerland). The assay was performed according to the manufacturer's instructions. Briefly, cells were labeled with BrdU labeling solution for 4 h, and the microplate was subsequently centrifuged at $300\times g$ for 10 min. The labeling medium was removed, and the cells were completely dried and fixed with the provided fixing solution for 30 min at room temperature. Subsequently, anti-BrdU-peroxidase solution was added to each well for 90 min at room temperature. Then, the antibody conjugates were removed, and the cells were washed three times with the provided washing solution. After treatment with the substrate solution, the reaction was stopped with 1 M sulfuric acid, and optical density (OD) was measured at 450 nm and 690 nm (reference wavelength) by a microplate reader (SPECTROstar NANO, BMG LABTECH).

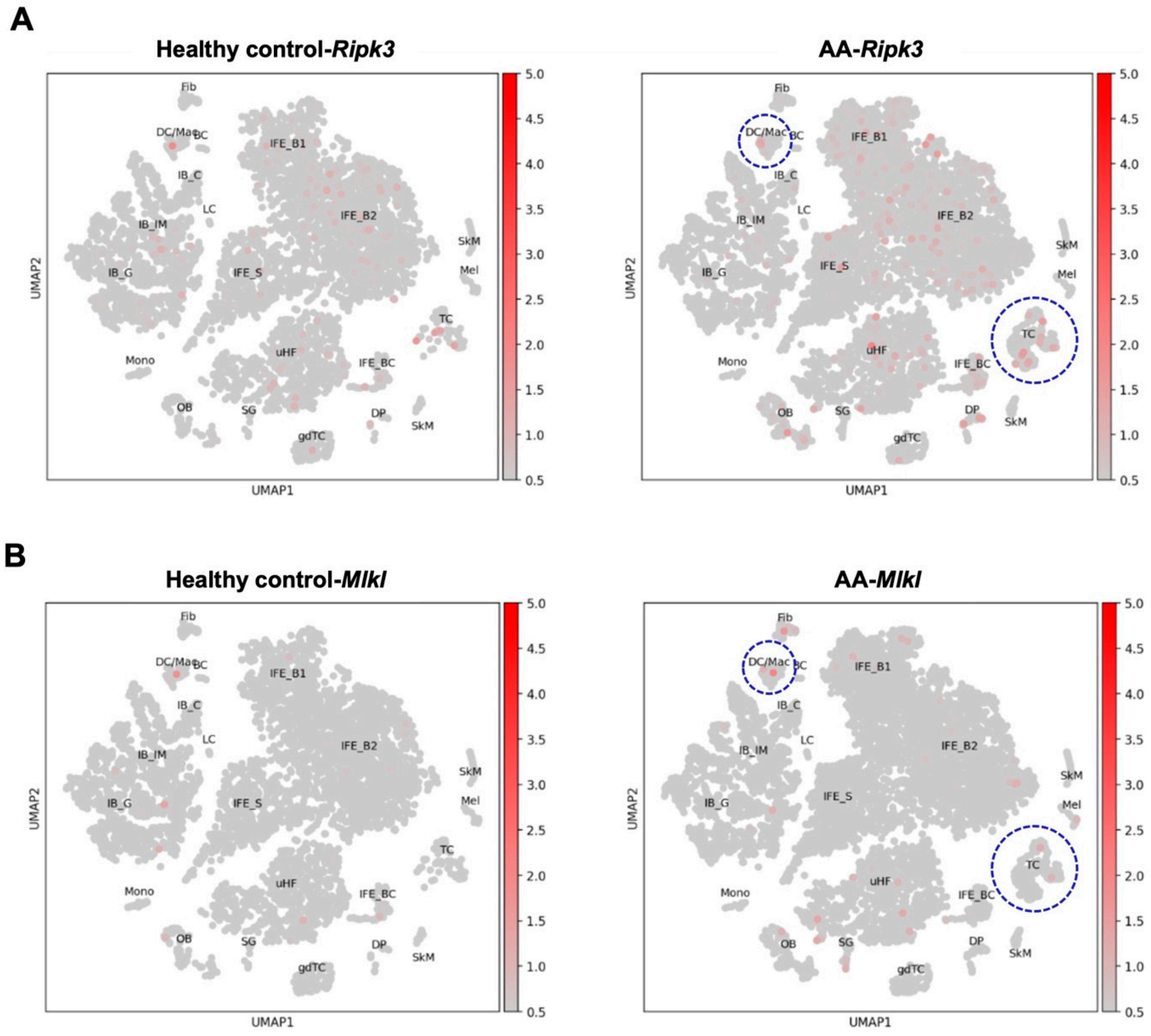

Figure S1. Expression profiles of *Ripk3* and *Mlkl* in the AA mouse model. (A) UMAP plot of *Ripk3* expression in control and AA mouse samples. (B) UMAP plot of *Mlkl* expression in control and AA mouse samples. The blue dotted line circle indicates the DC/Mac and T cell cluster.

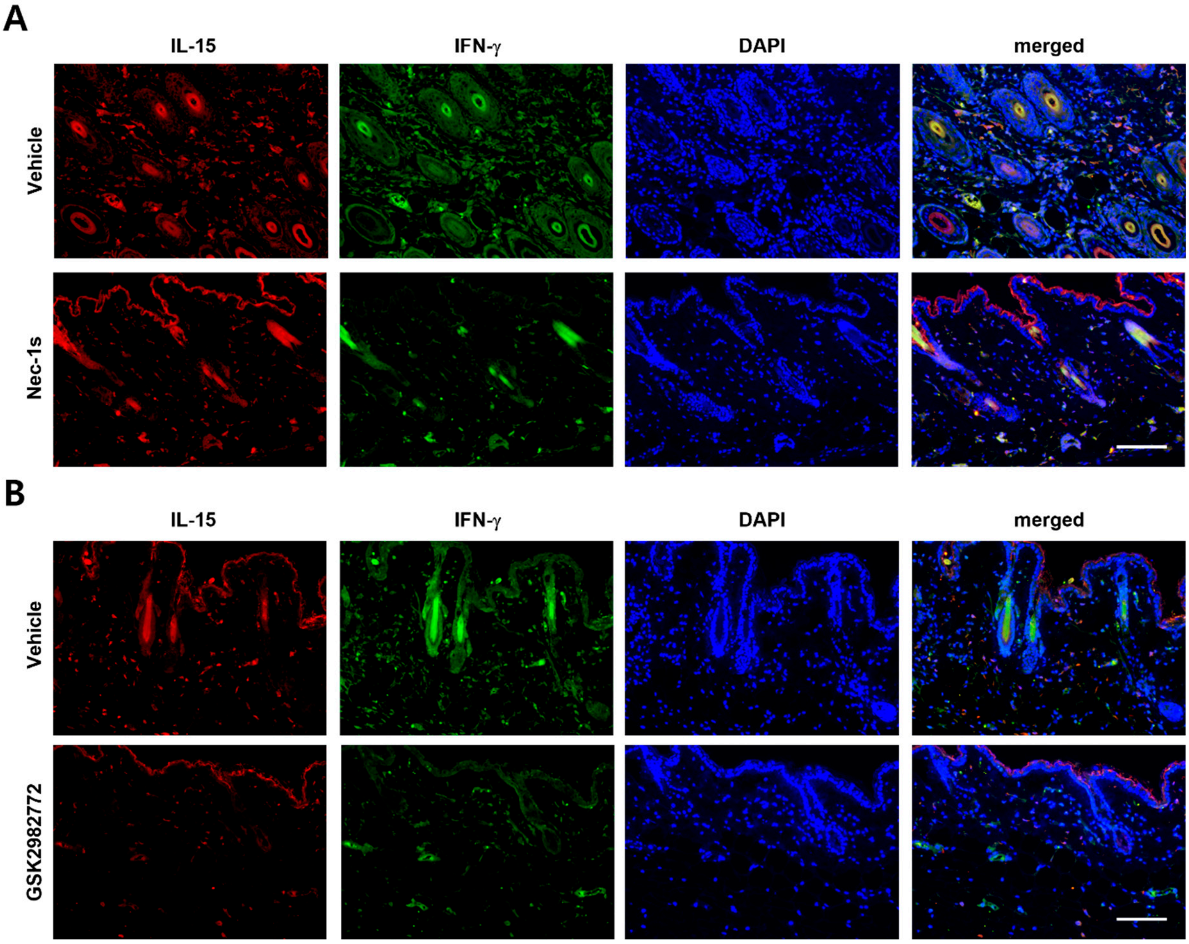

Figure S2. Effects of RIPK1 Inhibitors on IL-15 and IFN-γ expression in the AA onset model (A and B) Representative immunofluorescence image of skin section stained with IL-15, IFN-γ, and DAPI for nuclei. Scale bar: 100 μm.

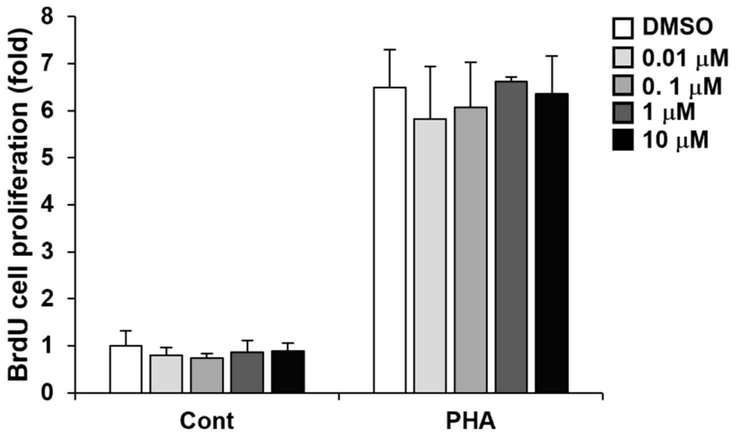

Figure S3. The effect of RIPK1 inhibitor in T cell activation. The effect of PBMC proliferative activity. PHA-stimulated PBMCs were treated with different concentrations of Nec-1s for 48 h. Cell proliferation was determined via BrdU assay.
